# 

**Published:** 1917-11-10

**Authors:** 


Rates of Subscription: Twelve months, 6/6; Six months, 4/-; three months, 2/-, post free to any address in the United Kingdom. Abroad, Twelve months, 8/8; Six months, 5/-; Three months, 2/6, post free. THE HOSPITAL "Index" to Volume LXI., October 1916 to March 1917, is now ready, price 2½d. per copy, post free. Address The Manager, The Hospital, "The Hospital" Building, 28/29 Southampton Street, Strand, London, W.C. 2.

### Medical Appointments

THE following house appointments have been made at St. Thomas's Hospital : *Casualty Officers and Resident Anæsthetists.*—F. C. Odling, B.A. Cantab., M.R.C.S., L.R.C.P.; J. G. McCann, M.R.C.S., L.R.C.P.; C. V. Isard, M.R.C.S., L.R.C.P.; H. I. Marriner. *Resident House Physicians*—W. E. Le Gros Clark, M.R.C.S., L.R.C.P.; J. S. Eloff, M.R.C.S., L.R.C.P.; E. A. Gibb, B.A. Cantab., M.R.C.S., L.R.C.P.; H. B. Russell, M.R.C.S., L.R.C.P. *Resident House Surgeons.*—T. F. M. Dilworth, M.B., B.Ch., B.A.O., N.U.I.; A. H. Hilmy, M.R.C.S., L.R.C.P., F. R. G. Hief, B.A. Cantab.; R. Calvo.—*Resident House Surgeon to Block 8.*—R. A. Walker.—*Resident Obstetric House Physician.*—F. N. Reynolds, M.R.C.S., L.R.C.P.

### Hospital Secretary Killed in Action.

WE regret to announce the death of the secretary of Worthing Hospital, Lieut. Arthur H. Tucker, Royal Sussex Regiment, attached to the King's Royal Rifles, who was killed in action in France on October 16. Before entering the Army Mr. Tucker practised as an architect in Worthing, which work he combined with his hospital duties. During Lieutenant Tucker's absence his wife has been carrying on his secretarial duties at the hospital.

### Sister Monk's Memorial Reredos.

We regret that an article of great interest to the nursing profession is unavoidably held over until next week's issue.

### Matrons' and Sisters' Appointments.

District Cottage Hospital, Ottery St. Mary.—Miss E. G. Newton has been appointed matron. She was trained at the Great Northern Central Hospital, London, and has been night sister at Newbury District Hospital and sister at Tiverton General Hospital.

Moorheads Hospital, Dumfries.—Mrs. Charlotte Jeffroy has been appointed matron. She was trained at the Crichton Royal Institution, Dumfries.

Kent and Canterbury Institute, Canterbury.—Miss M. I. Upton has been appointed superintendent. She was trained at St. Bartholomew's Hospital, E.C., and has been assistant superintendent of the Devon County Nursing Association and superintendent of the Taunton District Nurses' Home.

Isolation Hospital, Tivington, near Minehead.—Miss Isobel Llewellyn has been appointed matron. She was trained at the Western Fever Hospital, Fulham; since being charge nurse and temporary matron at Mastin Moor Hospital, Derbyshire, and matron at the East Ashford R.D.C. Sanatorium, Willesborough, Ashford.

Beckett Hospital, Barnsley.—Miss H. W. Phillips has been appointed casualty sister. She was trained at the Manchester Children's Hospital, Pendlebury, and at Addenbrooke's Hospital, Cambridge.

Borough Fever Sanatorium, Cambridge.—Miss Gertrude Praegar has been appointed sister. She was trained at University College Hospital and at the South-Western Fever Hospital, Stockwell. She has been in charge of wards in hospitals under the M.A.B. and at the Isolation Hospital, Norwich, and has been night superintendent at the Stamford, Rutland, and General Infirmary.

Borough Hospital, Hyde, Cheshire.—Miss Mary C. Jones has been appointed sister. She was trained at Brompton Hospital for Consumption, later being staff nurse there and at Brompton Sanatorium.

City of Birmingham Open-Air Sanatorium, Salterley Grange, Cheltenham.—Miss Margaret M. Grace has been appointed sister. She was trained at St. Mary's Hospital, Paddington, W.

Derbyshire Royal Infirmary.—Miss Lilian Smedley and Miss Hamilton-Swim have been appointed sisters. Miss Smedley was trained at the Westminster Hospital, London, and has been holiday sister there. Miss Hamilton-Swim was trained at St. Helen's Hospital, Lancashire, later being sister there, and at Warrington War Hospital.

Royal Victoria and West Hants Hospital, Bournemouth (Boscombe Branch).—Miss Florence Snow has been appointed sister. She was trained at Poplar Hospital for Accidents, where she was later sister, which post she has held also at the Royal Albert Hospital, Devonport. She has been sister under the Q.A.I.M.N.S. Reserve for two and a half years.

Tynemouth Union Hospital.—Miss Isabel Lee, Miss Maud Greenhow, and Mrs. G. Hedley have been appointed ward sisters. Miss Lee was trained at Hartlepool Union Hospital, where she was later staff and charge nurse; Miss Greenhow was trained at Newcastle Union Infirmary, and Mrs. Hedley at Sunderland Union Hospital, having since been staff nurse at Stockton-on-Tees Union Hospital and at the War Hospitals at Birmingham and Bradford.

**NOTE.—Particulars of Appointments for insertion in this column will be welcomed.**

### The Royal Hospital for Incurables.

The annual meeting of the Royal Hospital for Incurables will take place on November 30, at 11.30 a.m., at the Cannon Street Hotel. The date was stated in error in our issue of last week (page 88) to be November 2.

